# Healthy Eating Policy: Racial Liberalism, Global Connections and Contested Science

**DOI:** 10.1007/s41055-022-00111-5

**Published:** 2022-10-28

**Authors:** Christopher Mayes

**Affiliations:** Alfred Deakin Institute of Citizenship and Globalisation, Deakin University, 75 Pigdons Rd, Waurn Ponds, 3216 Australia

**Keywords:** John Rawls, Colonialism, Racial liberalism, Nutrition science, Global food system, Expertise

## Abstract

The challenges to designing and implementing ethically and politically meaningful eating policies are many and complex. This article provides a brief overview of Anne Barnhill and Matteo Bonotti’s *Healthy Eating Policy and Political Philosophy: A Public Reason Approach* while also critically engaging with the place of racial justice, global interconnectedness, and debates over science in thinking about ethics and politics of public health nutrition and policy. I do not aim to burden Barnhill and Bonotti with the responsibility to fully address these issues, but considering the interconnection of these issues and the ever pressing effects of climate change on local and global food systems, we collectively need to turn to these difficult and pressing questions about what a just food system looks like, what concerns are centred, and who is left out. I group these engagements with Barnhill and Bonotti under three headings: racial liberalism, global food system, and contested nutrition science. I conclude with some remarks about locality.

## Introduction

The entanglement of the COVID-19 pandemic, Black Lives Matter movement, and debates over scientific expertise in areas of climate science and epidemiology has led many professional societies and academic disciplines to re-think their history, purpose, and future (Eichelberger et al. 2016; Hardeman et al. 2020; Russell 2021; Morabia 2020; Bond et al. 2020). Scholars working on food, and how we nourish ourselves and each other, have also turned to consider these intersecting crises(Lee and Houston 2020; Stead and Hinkson 2022; Garth and Reese 2020). While the culmination of each of these crises may have a unique intensity, they are not new and have much longer histories. It is in the midst of these crises that Anne Barnhill and Matteo Bonotti published *Healthy Eating Policy and Political Philosophy*. It was not a stated objective of the book to specifically address the issues just described, but its argument is not irrelevant to them either. As I read this book my news and social media feeds were filled with examples of the interconnection of racial, health, and environmental injustices. There was also the bizarre phenomenon of people hoarding toilet paper, which I directly experienced but did not participate in, I swear. So, with all this going on around me it was hard not to read *Healthy Eating Policy and Political Philosophy* in light of these concerns and test its thesis against them.

At the outset I want to commend Barnhill and Bonotti for producing an impressive work; a welcome addition to the growing literature on the ethics and politics of public health nutrition and policy. A central aim of the book is “to develop a ‘public reason approach’ to healthy eating policy: to consider how a dominant and fruitful approach in political philosophy (public reason) applies to healthy eating efforts” and to create a tool “that can be used in the assessment of actual healthy eating efforts” (Barnhill and Bonotti 2021, 9). The “dominant approach” they use is John Rawls’ idea of public reason as a means of deliberation in liberal societies. As such, the focus of the book is on policymakers and health workers in middle- and high-income liberal democracies such as the United States, Britain, Australia, and so on. This book achieves the stated goal of providing a liberal political philosophy account of healthy eating policy. Furthermore, I believe it succeeds in giving people familiar with health and food policies an introduction into key arguments in liberal political philosophy, as well as offering a clear example to political philosophers of how to enter current public policy debates. However, I do have some critical comments specifically about the approach of the book, as well as some comments regarding next steps in thinking about ethics and politics of public health nutrition and policy. I group these comments under three headings: racial liberalism, global food system, and contested nutrition science. I conclude with some remarks about locality.

### Racial Liberalism

The entanglement of diets, food, and health with race and politics has a long a complex history. Only a few pertinent examples can be addressed here. The work of historian Rebecca Earle shows how food and diet were crucial components of European colonialism, nationalism, and capitalism (Earle 2010, 2012, 2019). Based on the humoral theory of health, food was of central importance for European self-understanding of their difference to other societies, cultures, and bodies (Earle 2010, 690). Rather than understanding bodies as fixed, they were understood as permeable and subject to transformation if exposed to new diets, climates, or environments (Meloni 2019). Hence sixteenth century Spanish colonisers in Latin America believed their complexion and bodily constitution would transform into that of the Amerindians if they adopted their diet. The reverse was also believed to be true so they encouraged Amerindians to “adopt the healthy diet of Europeans, for this in itself would improve their level of civility”(Earle 2010, 709). Although based on soft hereditarian ideas rather than humoralism, similar dynamics and anxieties about diet and race can be observed in nineteeth and twentieth century Australia. In the 1920s explicitly racialized nutrition programs were deployed in Australia to prevent the white colonisers moving into the tropics from becoming ‘native’ (Anderson 2002, 152). Prior to this, the protectorate or mission system in Australia, which operated from 1860 to 1960s, coercively aimed to get Aboriginal and Torres Strait Islander peoples to adopt a British way of life and diet with the dual objective to “civilise” and improve health (Rowse 1998; Boucher 2015). In Canada, during the 1940s and 50s, leading nutrition scientists conducted brutal experiments on Aboriginal children in residential schools to assist the “so-called transition from ‘traditional’ to ‘modern’ foodways” (Mosby 2013: 170). In the US, there were similar programs to erase or transform Indigenous foodways and sovereignty (Whyte 2017). Black foodways have also been the target of US public health programs (Reese 2019). For instance, the complex history of soul food and its contemporary denigration as unhealthy and the cause of disease and early death in Black communities distracts from structural determinants of health and calls for racial justice (Hassberg 2020: 85–86; Whitehead 1992; Hall 2020).

These are just a few examples that show how food, race, colonialism, and dietary guidance have a long and troubled history. The effects of these histories cannot be quarantined to the past. They continue to shape contemporary public health dietary guidance in settler-colonies such as Canada, United States, and Australia where Black, Indigenous, and peoples of colour continue to be the targets of public health policies and practices designed and implemented by predominantly white people and organisations (Delbridge et al. 2022). As such, the communities that endured colonialisation, slavery, and dispossession, often due to agriculture and food production (Mayes 2020; Whyte 2017), continue to endure racialized nutrition policies.

While Barnhill and Bonotti do not go into the detail of these histories, they are not ignorant of the effects of racism in health and nutrition today. At a number of points across their analysis they refer to structural racism and the way racialized communities have been targeted by public health policies (Barnhill and Bonotti 2021, 8, 60–62, 204). To their credit they give a lot more attention to these dynamics than many other books on ethics and politics of healthy diets. Marion Nestle’s highly-cited book *Food Politics*, for example, has nothing to say about racism except for one brief aside to the Black Caucus’ criticism that US dietary guidelines on dairy consumption represent a racial bias (Nestle 2007, 73). However, the question remains: is liberal political philosophy able to adequately attend to these histories of racism, colonialism, and their continued role in shaping the public sphere?

Barnhill and Bonotti allude to this problem in noting “Rawls’ own theory of justice was silent on structural injustice”, yet they contend that “his conception of public reason allows conceptions of justice that speak to structural justice” (Barnhill and Bonotti 2021, 158). This, however, is a live debate among Rawlsian political philosophers (Matthew 2017; Shelby 2013; Reidy 2022). The late Charles Mills – the Caribbean-American political philosopher who died in 2021 – argued that racism is baked into liberal political philosophy (Mills 2017). He particularly focused on Rawls, also going back through the liberal tradition to Locke, to argue that liberalism takes the white male experience of the world as the norm and that public reason is shaped by those who have been allowed to appear in public and who have been considered reasonable (Mills 2009, 2017). Mills argued that the historical formation of the social reality on which liberalism is built has been racialised (and gendered). By this Mills meant that race and structural injustice is not something that Rawls merely forgot to address, but his ignorance was part of his method (Mills 2007). Mills called this “white ignorance”, which other scholars such as Linda Martín Alcoff (2015) have used to highlight the way not knowing is a politically deployed to enable the continuation of the status quo. In contrast, Mills, Alcoff and others (Russell 2016; Taylor 2013) contend that the historically formed ontology and epistemology of political liberalism needs to be fundamentally re-thought with racial justice placed in the centre (Mills 2007). That is, rather than being ignorant or “blind” to race and histories of racism, Mills argued that race needs to be admitted into liberalism as a socio-political reality. So, what relevance does this have here?

As noted, food and dietary guidance have histories that are entwined with and shaped by racial liberalism. Racial liberalism has justified much of the British colonial enterprise in the US, Australia, Canada, New Zealand, and many of the other middle-and-high income liberal democracies that are the target of Barnhill and Bonotti’s analysis. Racial liberalism also shaped the legal constitutions of these societies. Furthermore, agriculture and food production had a dual function in securing the “frontiers” for the settler-colonial population as well as replacing the foodways and dietary patterns of Indigenous populations (Campbell 2020), thereby making them dependent on colonisers (Mayes 2018).

So, what would admitting race as a socio-political reality look like? By way of comparison, Health in All Policies (HiAP) is a popular public health concept used to emphasise the need for all social and public policies to consider health implications (Baum et al. 2014; Puska 2007). Following the lead of critical philosophers of race (Russell 2016), and Indigenous-led health researcher programs (Watego et al. 2021), perhaps we need to think about racial justice in every policy and consider what that would look like in relation to food, health, housing, and educational policies. Each of these areas intersect with each other and have long and troubled histories of contributing to racial injustices in middle- and high-income liberal democracies. A political liberalism that takes on Mills’ critique and proposals could provide valuable guidance for such an approach. Barnhill and Bonotti’s approach may have the flexibility to do this, but the Rawlsian approach needs to substantially attend to the critique put forward by Mills and others.

### Global Food System & Capitalism

In recent years the interconnection of the global food system, the power of transnational food corporations, and role of international governance has become increasingly apparent (Stead and Hinkson 2022; Lang and Heasman 2012; Rose 2013; McDonald 2010). Yet Barnhill and Bonotti carefully mark the scope of their analysis to the nation-state. They note that while “International health organizations and bodies have elevated healthy eating efforts as a global priority”, they are only focusing “on healthy eating efforts in liberal democracies, and particularly in those that are high-income countries” (Barnhill and Bonotti 2021, 9). While it makes sense to contain their analysis at the national level, the complex interconnections of the global food system make this difficult. As noted, international health organisations shape global and national nutrition guidance, just as transnational food corporations, global supply chains, and free trade agreements shape diets, labour, and migration movements (Stead and Hinkson 2022; Lang and Heasman 2012; Rose 2013; McDonald 2010; Gleeson and Friel 2013; Friel et al. 2013). So, while it is true that most nations develop and design their own dietary guidelines and therefore it makes sense for Barnhill and Bonotti to focus on this policy process. It is also true that international trade policies partly determine the kinds of policies that can be implemented at the national level. More on this shortly.

Related to their focus on the nation-state is their use of “citizen” and “citizenship”. In a footnote they qualify their use of citizenship in saying, “Throughout the book, we use the term ‘citizen’ broadly to also include those who reside in a country and are affected by its laws but are not legal citizens of that country” (Barnhill and Bonotti 2021, fn pg 4). Framing their analysis in terms of citizenship, even in this qualified way, doesn’t account for the global aspect for contemporary food and health systems. What about those residing in one country but are affected by the food and health policies of another country? For instance, in the US turkey tails are removed prior to sale due health and culinary reasons (Carolan 2017). The tails are very fatty and not considered appetizing by American consumers. Yet, since the 1950s US poultry farmers have developed an export market for turkey tails to Samoa. The Samoan government banned the importation of tails in 2007 due to concerns about obesity (Thow et al. 2010), but when they joined the World Trade Organisation in 2013, they were forced to lift the ban despite public health concerns (Carolan 2017). Similarly, peoples throughout the two-thirds world and global south have had their local food systems significantly disrupted by structural adjustment programs emanating from western liberal democracies (Moseley et al. 2010). A final example is the incursion of industrial fishing fleets, backed by Chinese and EU investors, into the waters of small-scale fishing communities in low-income countries, thereby threatening livelihoods and food security (Okafor-Yarwood et al. 2022).^1^ While restricting their analysis to Western liberal democracies makes sense in terms of scope, it is difficult to contain the ethics and politics of healthy eating policies to the nation and its citizens when the effects of food corporations and food and agricultural policies are not limited to those jurisdictions.

Finally, Barnhill and Bonotti recognise this when commenting on dietary advice and environmental sustainability saying, “not all healthy dietary patterns could be widely adopted in an environmentally sustainable way” (Barnhill and Bonotti 2021, 19). They note that the US recommendation to consume more fish “could deplete global fish supplies and threaten fishers’ livelihoods” and therefore “governments keen to promote healthier dietary patterns should consider pursuing both goals simultaneously, through efforts that identify and promote dietary patterns that are both sustainable and healthy” (Barnhill and Bonotti 2021, 19). Thus healthy eating policies do not only need to consider global influence of food corporations, but also shared ecologies and environments.

My point here isn’t that focusing on national level policies is unimportant or that Barnhill and Bonotti’s public reason approach needs to have a solution for these complex and globally interconnected problems. Rather my point is to increase the burden of thinking about ethical healthy eating policies in western liberal democracies like the US, Canada, Australia and so on. Not only do these nations have obligations to their own “citizens”, but they have had an inordinate role in shaping, or more accurately disrupting, global diets and food cultures through their roles in designing global food and agricultural trade policies as well as being home to many transnational food corporations (Stead and Hinkson 2022). Mirroring Rawls’ extension of his theory of justice from domestic to international politics (Rawls 1999), it will be interesting to see the ways Barnhill and Bonotti’s analysis can be extended to address the healthy-eating policy among peoples.

### Contested Nutrition Science

The final point I wish to discuss is the contested reality of nutrition science and its application in dietary guidance. I am particularly interested in claims about causal relations between food choices and certain health or disease outcomes. Barnhill and Bonotti note that “different empirical and epistemic assumptions often underlie healthy eating efforts” particularly “those concerning the causal relationship between eating and health (or lack thereof)” (Barnhill and Bonotti 2021, 69). There is often fierce debate among scientists as well as those seeking to use the science as an evidence base for nutrition guidance (Katz and Meller 2014; Mayes and Thompson 2014; Scrinis 2013; Hite 2018).

The EAT-Lancet commission (Willett et al. 2019), for example, sought to produce a diet that would guarantee health for 10 billion people while ensuring the sustainability of the planet (Barnhill and Bonotti 2021,18). Their report has garnered significant support among public health advocates and environmentalists, yet it has also generated backlash (Zagmutt et al. 2019; Kaiser 2021), specifically around the recommendation for a primarily plant-based diet to secure planetary health. Critics argue that the commission misrepresent or overstate the scientific evidence regarding the place of animal-protein in a healthy diet (Leroy and Cofnas 2020; Leroy and Hite 2020). One approach to defend the report and its scientific basis has been to cast these critics as biased and influenced by meat and livestock industries (Garcia et al. 2019). It is undeniable that those in the meat and livestock industry are highly critical of suggestions that people need to consumer less meat (Clare et al. 2022). However, it is a convenient response that does not account for the complexity and contested relations among nutrition science, dietary guidance, and long-term health outcomes for individuals and populations across different cultural and socio-economic contexts (Overend 2020; Biltekoff 2013; Mayes and Thompson 2015). These debates cannot be resolved here, my point is simply that nutrition science *is* contested and that disagreements over dietary guidance are not reducible to “bad science”, corruption, or bias.

Barnhill and Bonotti note the debates over nutrition science, yet they maintain that in liberal democracies’ there is generally cause for high level of trust in science as a reliable source of truth (Barnhill and Bonotti 2021, 157). As such, they believe devices such as nutrition facts labels can provide “objective nutrition information” (Barnhill and Bonotti 2021, 95) and are “necessary to ensure that consumers are fully aware of the implications of their actions and voluntarily choose to perform them (even if these actions are “bad” or dangerous for them)” (Barnhill and Bonotti 2021,92). However, a further dimension to the contested nature of nutrition science and dietary advice is the social and political contexts in which it is applied. The right or best approach to nutrition food labelling is hotly debated among policymakers, politicians, consumer groups, public health nutritionists and industry representatives (Silverglade and Heller 2010).

While I have previously argued that nutrition (and ethics) labels do not offer objective information (Mayes 2014a, 2014b, 2016), I do think Barnhill and Bonottio’s model can help address a more significant problem, namely, the capture of political legitimatising mechanisms. In Australia, for example, healthy star food labelling system was developed through a supposedly deliberative process that included consumers, public health, industry and health policy representatives. While politicians claimed this process included all parties and thereby the outcomes had political legitimacy, it was clear to many observers in the media and academy that the process had been captured by industry interests (Sacks 2014; Mayes 2014b).

A final dimension to the contested nature of nutrition science is that our tastes, desires, and pleasures change over time. As Barnhill and Bonotti note, “food marketing strategies that have, over time, altered our gustatory preferences (e.g. we now favour sweeter and saltier food)” (Barnhill and Bonotti 2021, 48). They contend that the influence of food marketing in shaping dietary preferences and desires inhibits the possibility for individuals to critically reflect on “their unhealthy dietary patterns’ and their long terms health implications” (Barnhill and Bonotti 2021, 42). The influence of food marketing (and food science) in changing our culinary preferences is obviously true. However, it is important to recognize that public health nutrition and health policies have both changed the way the public understands food and its relation to health. A number of studies highlight how public health nutrition campaigns have done a lot of marketing work for food corporations by making a public receptive to “vitamins”, “high fibre”, “super-foods”, “probiotics” and so on (Gardner 2006; Scrinis 2013; Mayes 2014a; Biltekoff 2013; Loyer 2016). Furthermore, the rise of wellness culture and healthism over the past 50 years has created an expansive and lucrative health food market that draws on nutrition science to modify and transform tastes and desires (Crawford 1980; Hite 2018; Mayes 2015; Overend 2020).

Considering these trends, it is difficult to conclude that there is a neutral or objective healthy diet abstracted from the social, commercial, and historical influences on dietary experience (Kamminga and Cunningham 1995). This is simply to say that even within the context of nutrition science, but certainly beyond it, the notion of a “healthy diet” or even a “healthy food item” change over time due to social and cultural values as well as scientific, political, and commercial influences.

### Local Conclusions

Barnhill and Bonotti’s book offers useful ways to think through and address some of the issues I have raised here, and others are beyond its scope. In concluding, I think their model offers possibilities for local communities to define their own foodways and dietary guidance. As Barnhill and Bonotti note, “healthy eating efforts for some groups should emphasize not only eating in ways that reduce disease risk, but also eating in ways that promote health as they understand it” (Barnhill and Bonotti 2021, 66). While the issues I mentioned in the introduction, as well as those Barnhill and Bonotti identify, are global or national in scope, many of the solutions require local ideas, participation, and action. Allowing people and communities the opportunity to discuss and define health is lacking in a lot of dietary guidance. This is particularly concerning as food intersects with people’s lived experience of health and wellbeing, which is often believed to be something more than extending or reducing bio-physical lifespan. Barnihill and Bonott’s emphasis on justice in designing food policies provides a good platform from which to start these conversations.

## Footnotes

1 I wish to acknowledge the anonymous reviewer who drew my attention to this example and its further significance due to the intersection of climate justice and food justice for these communities.
